# Fractal correlation property of heart rate variability in response to the postural change maneuver in healthy women

**DOI:** 10.1186/1755-7682-7-25

**Published:** 2014-05-15

**Authors:** Ana CA de Souza, José R Cisternas, Luiz Carlos de Abreu, Adriano L Roque, Carlos BM Monteiro, Fernando Adami, Luiz Carlos M Vanderlei, Fernando H Sousa, Lucas L Ferreira, Vitor E Valenti

**Affiliations:** 1 Departamento de Morfologia e Fisiologia, Faculdade de Medicina do ABC, Av. Príncipe de Gales, 821, Santo André, SP 09060-650, Brazil; 2 Departamento de Fisioterapia, Faculdade de Filosofia e Ciências, Universidade Estadual Paulista, Marília,UNESP, Av. Higyno Muzzi Filho, 737, Marília, SP 17.525-900, Brazil; 3 Programa de Pós-Graduação em Fisioterapia, Faculdade de Ciências e Tecnologia, Universidade Estadual Paulista, UNESP, R. Roberto Simonsen, 305, Presidente Prudente, SP 19060-900, Brazil; 4 Universidade Estadual Paulista, UNESP, Av. Eng. Luiz Edmundo Carrijo Coube, 14-01, Bauru, SP 17033-360, Brazil

**Keywords:** Autonomic nervous system, Cardiovascular system, Nonlinear dynamics, Physiology

## Abstract

**Background:**

We evaluated the effects of the PCM on the fractal analysis of the HRV in healthy women

**Method:**

We evaluated healthy women between 18 and 30 years old. HRV was analyzed in the time (SDNN, RMSSD, NN50 and pNN50) and frequency (LF, HF and LF/HF ratio) domains as well as short and long-term fractal exponents (alpha-1 and alpha-2) of the detrended fluctuation analysis (DFA). HRV was recorded at rest for ten minutes at seated rest and then the women quickly stood up from a seated position in up to three seconds and remained standing for 15 minutes. HRV was recorded at the following time: rest, 0–5 min, 5–10 min and 10–15 min during standing.

**Results:**

We observed decrease (p < 0.05) in the time-domain indices of HRV between seated and 10–15 minutes after the volunteer stood up. The LF (ms^2^) and HF (ms^2^) indices were also reduced (p < 0.05) at 10–15 minutes after the volunteer stood up compared to seated while the LF (nu) was increased at 5–10 min and 10–15 min (p < 0.05). The short-term alpha-1 exponent was increased (p < 0.05) at all moments investigated compared to seated. Increase in the properties of short-term fractal correlations of heart rate dynamics accompanied by a decrease in the parasympathetic modulation and global HRV was observed in response to the postural change maneuver.

**Conclusion:**

We suggest that fractal analysis of HRV is more sensitive than frequency and time-domain analysis of HRV during the postural change maneuver.

## Background

Heart rate variability (HRV) is a conventionally accepted term to describe the fluctuations in the intervals between consecutive heartbeats (RR intervals), which are related to influences of the autonomic nervous system (ANS) on the sinus node [1,2]. There are several methods to measure HRV [3-6].

Nonlinear methods that analyze HRV have received great attention. It is suggested that mechanisms involved in cardiovascular regulation probably interact between each other in a nonlinear style [7,8]. In this sense, a well-recognized method used for nonlinear analysis is detrended fluctuation analysis (DFA). This method quantifies the absence or the presence of fractal correlation properties of the RR intervals [7,9]. Fractal indices were indicated to detect slight changes in the RR intervals dynamics better than conventional spectral analyses, including time and frequency domains. Furthermore, changes in fractal correlation properties of long- and short-term dynamics of HRV helps clinical professionals to avoid disease development and identify autonomic impairment [10].

The examination of autonomic cardiovascular tests may provide important information about appropriate function of the ANS as well as functional capacities of effectors (heart and vessels) and other associated structures. It may also be used for cardiovascular system control investigation in healthy subjects, in adult patients with different diseases and for the diagnosis of autonomic dysfunction. One commonly cardiovascular test in clinical practice used is the postural change maneuver (PCM), which is based on the measurement of heart rate reflex changes in response to adequate stimuli [11].

Although previous studies investigated spectral analysis of HRV in response to the PCM [12,13] and Tulppo et al. [10] previously reported the behavior of the alpha-1 during the passive tilt test, it is not clear the responses of the fractal analysis of HRV in the active PCM. In addition, the knowledge of physiological responses induced by autonomic tests is relevant to provide further information regarding autonomic cardiac regulation and autonomic dysfunction diagnosis [11]. The main purpose was to gain insight into the physiological background for fractal and complexity characteristics of HR dynamics.

The responses caused by the passive orthostatic test are well documented in the literature [12,13]. However, the PCM performed from seated to standing is not well understood regarding the responses of the fractal exponents. Moreover, it is not clear in the literature how long the autonomic nervous system spends to induce sympathetic and parasympathetic changes immediately after the change of position. Thus, in order to provide methodological information regarding this autonomic test, we aimed to investigate the effects of the PCM on fractal exponents through DFA in young women, as well as the time and frequency domains indexes of HRV.

## Method

### Study population

We analysed 11 apparently healthy student women aged between 18 and 30 years old. All volunteers were informed about the procedures and objectives of the study and, after agreeing, signed a consent form. All study procedures were approved by the Research Ethics Committee (REC) of the institution (case number.2011/382) and followed the Resolution 196/96 of the National Health Council.

### Non-inclusion criteria

We did not include women under the following conditions: body mass index (BMI) >35 kg/m^2^; systolic blood pressure (SBP) >140 mmHg or diastolic blood pressure (DBP) >90 mmHg (at rest); cardiac arrhythmias (atrial flutter or fibrillation, multiple ventricular or atrial ectopy, second or third degree atrioventricular block), smoking, left ventricular dysfunction, neurological or respiratory disorders and serious postural deviation in the chest such as severe scoliosis, kyphosis or hyperlordosis that could influence the respiratory pattern and auditory disorders.

### Initial evaluation

Before the experimental procedure, volunteers were identified by collecting the following information: age, gender, weight, height and body mass index (BMI). Anthropometric measurements were obtained according to Lohman et al. [14]. Weight was determined by using a digital scale (W 200/5, Welmy, Brazil) with a precision of 0.1 kg. Height was determined by using a stadiometer (ES 2020, Sanny, Brazil) with a precision of 0.1 cm and 2.20 m of extension. Body mass index (BMI) was calculated using the following formula: weight (kg)/height (m^2^). We also measured systolic and diastolic blood pressure and heart rate.

### Experimental protocol

Data were collected in our laboratory under controlled temperature (21°C–25°C) and humidity (50%–60%), and volunteers were instructed to avoid consuming alcohol, caffeine and substances that influence the ANS for 24 hours before evaluation. Data were collected between 8 and 12 AM. All procedures necessary for the data collection were explained to the individuals, and the subjects were instructed to remain at rest and to avoid talking during the data collection.

After the initial evaluation the heart monitor belt was then placed over the thorax, aligned with the distal third of the sternum and the Polar RS800CX heart rate receiver (Polar Electro, Finland) was placed on the wrist. The subject remained 10 minutes seated at rest with spontaneous breathing. After ten minutes the volunteers quickly stood up from a seated position in up to three seconds according to verbal command and remained standing for 15 minutes.

### HRV analysis

The R-R intervals recorded by the portable HR monitor (with a sampling rate of 1000 Hz) were downloaded to the Polar Precision Performance program (v. 3.0, Polar Electro, Finland). The software enabled the visualization of HR and the extraction of a cardiac period (R-R interval) file in “txt” format. Following digital filtering complemented with manual filtering for the elimination of premature ectopic beats and artifacts, at least 256 R–R intervals were used for the data analysis. Only series with more than 95% sinus rhythm was included in the study [15-17]. HRV was analyzed at four moments: seated rest with spontaneous breathing, 0–5 minutes, 5–10 minutes and 10–15 minutes at standing position. We evaluated the linear and non-linear indices of HRV. For calculation of the indices we used the HRV Analysis software (Kubios HRV v.1.1 for Windows, Biomedical Signal Analysis Group, Department of Applied Physics, University of Kuopio, Finland) [18,19].

### Linear indices of HRV

For HRV analysis in the frequency domain we used spectral components of low frequency (LF: 0.04 to 015 Hz) and high frequency (HF: 0.15-0.40 Hz), inabsolute (ms^2^) and normalized units and the ratio between components of low and high frequency (LF/HF). Spectral analysis was calculated using the algorithm of fast Fourier transform [15].

The analysis in the time domain was performed by means of SDNN (standard deviation of the average normal RR intervals), RMSSD (square root of the mean squared differences between adjacent normal RR intervals) and pNN50 (percentage of adjacent RR intervals with a difference of duration greater than 50 ms) [15].

### Fractal analysis of HRV

For the analysis of the fractal properties of the heart rate, detrended fluctuation analysis (DFA) was applied to a time series of the R–R intervals obtained from the participants. The procedure for the calculation of DFA is made up of the following steps:

The R–R series obtained experimentally is integrated using the expression [9,10]:

$$Y\left(k\right)=\sum_{i=1}^{k}\left[\mathrm{RR}\left(i\right)-\mathrm{RRave}\right]$$

in which Y(k) is the k-th term of the integrated series (k = 1, 2,…, N); R–R(i) is the i-th value of the R–R intervals; and R–R_ave_ is the mean of the R–R intervals of the original series, with N length:

$$\mathrm{RRave}=\frac{1}{N}\sum_{i=1}^{N}\mathrm{RR}\left(i\right)$$

The integrated time series is then divided into intervals with a length of n, (n = 1, 2,…, N). In each of these intervals, the local trend of the series is calculated by a straight line of minimum squares adjusted to the data. The y-coordinate of this straight line was denominated Yn(k). The integrated series was then detrended [Y(k)], subtracting the local tendency Yn(k) in each interval. For a given interval of size n, the size characteristic of the fluctuation for the integrated and detrended series is calculated by:

$$F\left(n\right)=\sqrt{\frac{1}{N}\sum_{K=1}^{N}\left[Y\left(k\right)-\mathrm{Yn}{\left(k\right)}^{2}\right]}$$

This procedure is repeated for all intervals of size *n*, thereby obtaining a relation between the mean of the fluctuations [*F*(*n*)] and the size of the intervals (*n*). A linear relation on a log–log graph indicates a scale exponent law, based on the following formula:

$$F\left(n\right)\approx \mathrm{n\alpha}$$

in which *α* is the scale exponent, which can be calculated by linear regression on a log–log graph (16). The following were calculated: short-term fractal exponent (alpha-1), corresponding to a period of 4 to 11 beats; long-term fractal exponent (alpha-2), corresponding to periods longer than 11 beats; and the alpha-1/alpha-2 ratio [20].

When α = 0.05, there is no correlation and the signal consists of white noise; if α = 1.5, the signal resembles random walk (Brownian motion); and if 0.5 < α < 1.5, there are positive correlations. If alpha is close to 1.0 it indicates a more complex (non-linear) system, if it reaches values above 1.0 the system tends to be less complex and linear.

### Statistical analysis

Standard statistical methods were used for the calculation of means and standard deviations. Normal Gaussian distribution of the data was verified by the Shapiro-Wilk goodness-of-fit test (z value >1.0). For parametric distributions we applied the ANOVA for repeated measures followed by the Bonferroni posttest (SDNN, alpha-2 and alpha-1/alpha-2). For non-parametric distributions we used the Friedman test followed by the Dunn’s test (RMSSD, pNN50, LF, HF, LF/HF and alpha-1). We compared the HRV indices between the four moments (seated rest *vs*. 0–5 min after the volunteers stood up *vs*. 5–10 min after the volunteers stood up *vs*. 10–15 min after the volunteers stood up). Differences were considered significant when the probability of a Type I error was less than 5% (p < 0.05). We used the Software GraphPad StatMate version 2.00 for Windows, GraphPad Software, San Diego California USA.

## Results

Table 1 shows the values for diastolic (DBP) and systolic blood pressure (SBP), heart rate (HR), mean RR intervals, weight, heightand BMI of the volunteers.

**Table 1:** Baseline diastolic (DAP) and systolic arterial pressure (SAP), heart rate (HR), mean RR interval, weight, height and body mass index (BMI) of the volunteers

**Variable**	**Value**
Height (m)	1.69 ± 0.05
Weight (kg)	55.7 ± 29
BMI (kg/m^2^)	22.4 ± 3
HR (bpm)	76.2 ± 10
Mean RR (ms)	764.2 ± 78
SAP (mmHg)	103.4 ± 15
DAP (mmHg)	73.3 ± 14

Mean + standard-deviation. m: meters; kg: kilograms; bpm: beats per minut; mmHg: millimeters of mercury.

Table 2 presents data related to the time-domain indices before and during standing. We noted that the SDNN, RMSSD and pNN50 indices were reduced between 10 and 15 minutes during standing (control vs. 10–15 min).

**Table 2:** Mean and standard-deviation for time-domain indices between before and after the orthostatic test

**Index**	**Control**	**0-5 min**	**5-10 min**	**10-15 min**	**p**
SDNN (ms)	45.4 ± 6	43.5 ± 15	38.2 ± 9	*34.1 ± 4	0.045
RMSSD (ms)	30.1 ± 11	18.5 ± 7	16.4 ± 9	*15.2 ± 4	0.0176
pNN50 (%)	12.3 ± 15	3.7 ± 3	2.9 ± 3	*2 ± 1	0.012

SDNN: standard deviation of normal-to-normal R-R intervals; pNN50: the percentage of adjacent RR intervals with a difference of duration greater than 50 ms; RMSSD: root-mean square of differences between adjacent normal RR intervals in a time interval. ms: millisecond. *p < 0.05: Vs. Control.

Table 3 displays results concerning the frequency-domain indices before and during standing. We observed that the LF (ms^2^) and HF (ms^2^) indices decreased, while the LF (nu) index increased between 10 and 15 minutes during standing (control vs. 10–15 min). The HF (nu) index tended to be reduced and the LF/HF ration tended to increase.

**Table 3:** Mean and standard-deviation for time-domain indices between before and after the orthostatic test

**Index**	**Control**	**0-5 min**	**5-10 min**	**10-15 min**	**p**
LF (ms^2^)	791 ± 460	611 ± 232	635 ± 340	*500.1 ± 243	0.032
HF (ms^2^)	595.5 ± 621	291 ± 300	203 ± 188	*192.1 ± 190	0.026
LF (nu)	65 ± 12	74.5 ± 12	*75.9 ± 17	*76.1 ± 13	0.04
HF (nu)	32 ± 11	27 ± 19	21.2 ± 19	24.8 ± 11	0.07
LF/HF	2.3 ± 2	4.9 ± 4	5.3 ± 3	5.4 ± 3	0.06

LF: low frequency; HF: high frequency; LF/HF: low frequency/high frequency ration. ms: milliseconds; nu: normalized units. *p < 0.05: Vs. Control.

Table 4 shows the behavior of the fractal analysis of HRV before and during standing. The alpha-1 values were significantly increased throughout the 15 minutes during standing. On the other hand, there were no significant changes with respect to the alpha-2 and alpha1-/alpha-2 ratio values during standing.

**Table 4:** Mean and standard-deviation for alpha-1, alpha-2 values and alpha-1/alpha-2 ratio before and after the orthostatic test

**Index**	**Control**	**0-5 min**	**5-10 min**	**10-15 min**	**p**
Alpha-1	1 ± 0.06	*1.3 ± 0.03	*1.4 ± 0.02	*1.4 ± 0.02	0.019
Alpha-2	0.89 ± 0.2	0.91 ± 0.2	0.95 ± 0.19	0.91 ± 0.3	0.5
Alpha-1/Alpha-2	1.53 ± 0.6	1.69 ± 0.5	1.7 ± 0.32	1.79 ± 0.8	0.06

*p < 0.05: Vs. Control.

## Discussion

This study was undertaken to evaluate the behavior of the fractal indices of HRV after the volunteer stood up, a test usually studied to investigate autonomic cardiac function [11]. Our focus was to better evaluate the mechanisms related to HR dynamics in response to the PCM. The short term scaling alpha-1 ranges between around 0.5 and 1.5, if alpha is close to 1.0 it indicates a more complex (non-linear) system, if it is above 1.0 the system tends to be less complex and linear, indicating a bad prognosis or inadequate physiology [20]. As a main finding, the alpha-1 value was increased the entire 15 minutes when the volunteers were standing, indicating a deviation of this property to a more linear way. This finding was accompanied with decrease in SDNN, RMSSD and pNN50 time-domain, increase in LF (nu) and decrease in HF (ms^2^) and LF (ms^2^) frequency-domain indices of HRV mainly at 10–15 minutes. On the other hand, we found no significant changes in the fractal analysis regarding alpha-2 and alpha-1/alpha-2 ratio.

Considering the time-domain indices of HRV, reduction in SDNN, RMSSD and pNN50 observed in our study correspond to reduction in global HRV and parasympathetic cardiac regulation, since SDNN is related to global HRV while RMSSD and pNN50 are associated with parasympathetic activity [3,21]. Moreover, frequency-domain indices of HRV were also changed in response to the PCM. We reported reduction in LF (ms^2^) and HF (ms^2^) while LF in normalized units (nu) increased 10–15 minutes after the change from seated to standing, indicating cardiac sympathetic activation in response to the PCM. A recent study investigated sympathetic and parasympathetic cardiovascular components in response to the orthostatic test in women with and without hot flushes [22]. The authors reported that a change from a supine to a standing position caused an increase in the LF power and a decrease in the HF power. Nonetheless, the time-domain indices of HRV were not evaluated in that study. The novelty of our study is that we analyzed the HRV indices each 5 minutes during 15 minutes after the volunteers quickly stood up. We observed more intense responses at 10–15 minutes after the postural change.

In our study we evaluated HRV in each five minutes in order to observe the responses induced with more detail immediately after the change from seated to standing. While the linear indices of HRV changed with more intensity at 10–15 minutes after the changed from seated to standing, we reported increased alpha-1 values during the 15 minutes after the subject stood up. The DFA technique is a modified root-mean-square analysis of a random walk, and it quantifies the presence or absence of fractal correlation properties in the time series. The fractal scaling of HRV is based on values ranging from 0.5 to 1.5, the closer the 1.0, the more complex (non-linear) the system, above or below 1.0 the system tends to be less complex and linear. In this context, the exponents do not present direct or indirect relationship with the sympathetic or parasympathetic system. In this method, a fractal-like signal results in an exponent value of; 1.0, a random signal results in a value of 0.5, and a strongly correlated signal behavior linear results in an exponent value of 1.5 [23]. Enhanced short-term fractal exponent values (alpha-1 around 1.4) reported in response to the PCM in our investigation indicated stronger correlation properties of short-term HRV during the active test compared with the baseline resting conditions. The deviation of alpha-1 to a more linear way indicates a reduced HRV at the moments investigated.

In relation to long-term exponents (alpha-2) and alpha-1/alpha-2 ratio, there was no change in response to the PCM. On the other hand, alpha-1 was changed in response to the PCM, as well as the time-domain indices of HRV. Tulppo et al. [24] evaluated the alterations in the alpha-1 fractal exponent and in the frequency-domain indices of HRV induced by the passive head-up tilt test and dynamic exercise in healthy male volunteers. Similar to our findings, they revealed increase in the LF (nu) index during exercise, however, no significant changes were observed during the passive tilt test. They also found reduction in the HF power in the passive tilt test. In addition, the authors revealed that the short-term fractal scaling exponent alpha-1 increased during low-intensity dynamic exercise and during passive head-up tilt test. Our results suggest that the PCM from seated to standing position presents similar or maybe more intense cardiac autonomic responses to the passive tilt test [24].

The increased alpha-1 exponent in response to the PCM in our study was parallel to decrease in the parasympathetic activity observed by a reduction in the HF (ms^2^) index, which corresponds to the respiratory modulation and is an indicator of the performance of the vagus nerve on the heart [21]. The interaction between the vagal and the sympathetic regulation of HR is usually controlled in a reciprocal mode, such as amplified activity of one system associated with a reduced activity in the other system [25]. Those reciprocal alterations in the vagal and in the sympathetic tome were observed in response to the PCM in our study. The HF power of R-R intervals decreased as evidence of withdrawal of vagal activity.

The reciprocal changes in autonomic regulation reported in our investigation resulted in stronger short-term fractal correlation expressed as an increased alpha-1 exponent value. The changes observed in the HF and LF in absolute units (ms^2^) was observed only 10–15 minutes after the volunteer stood up while the alpha-1 short-term exponent was significantly increased 0–15 minutes after the volunteer stood up. According to Tulppo et al. [24], fractal indices are able to detect slight changes in the dynamics of RR intervals better than conventional spectral analyses. Taken together, we suggest that the alpha-1 exponent is able to detect slighter physiological changes in the autonomic cardiac regulation compared to the time and frequency domain indices of HRV [10,24].

The time-domain indices of HRV were also accompanied by the increase in the alpha-1 short-term exponent. SDNN, RMSSD and pNN50 reduced at 10–15 minutes after volunteer stood up. The SDNN index represents the sympathetic and parasympathetic activity, however, it does not allow to distinguish whether HRV changes are due to increased sympathetic tone or the withdrawal of vagal tone [18,21]. The RMSSD and pNN50 indices correspond to the parasympathetic activity, since those indices are found from the analysis of adjacent RR intervals [18,21].

We found no changes regarding the alpha-2 exponent. This exponent corresponds to Brownian motion, it is thought to indicate the total loss of the response loops and collapse of the processes that regulate heart rate during pain, resulting in highly correlated fractal dynamics that spread into many time scales [10].

Changes in alpha-1 were reported during infusion of physiological doses of norepinephrine in healthy subjects [10]. According to Tulppo et al. [24], during norepinephrine infusion, the HF-power increased during the initial dose. On the other hand, HF and LF spectral components decreased during a high dose of atropine infusion and increased during a low dose of atropine infusion. Regarding SDNN, its mean values increased during noradrenaline infusion. During atropine infusion the SDNN index increased with low dose while it reduced with high dose. Accompanied with these changes, the fractal scaling exponent alpha-1 reduced progressively during the incremental doses of norepinefrine infusion. Conversely, this index increased during the high dose of atropine [26]. Nonetheless, our findings suggest the simultaneous increase in the sympathetic activity and decreased short-term fractal organization of HR (alpha-1 far from 1.0) in healthy humans without pharmacological manipulation of the autonomic cardiac regulation.

We provided detailed information in each five minutes indicating that reduced vagal activity and global HRV resulted in a loss or breakdown of the short-term fractal organization of HR, expressed as an increased alpha-1 value in response to the PCM. On the other, no expressive results were found for the long-term fractal alpha-2 exponent and alpha-1/alpha-2 ratio.

Regarding the fractal analysis of HRV, when alpha1 is 0.05, it corresponds to no correlation and the signal consists of white noise, if it is 1.5, the signal resembles random walk (Brownian motion); and if 0.5 < α < 1.5, there are positive correlations, i.e., the closer to 1.0 the more complex the system and better for the organism physiology [24]. In this sense, we observed reduced parasympathetic regulation of the heart in response to the active orthostatic test through analysis of the time and frequency domain accompanied by values of alpha-1 higher than 1.0 and far from 1.0, indicating that the system is overcharged in response to this test. We suggest that alpha values higher and far from 1.0 represent decreased parasympathetic and increased sympathetic modulation of the heart.

Our study presents some points that should be raised. The study of Tulppo et al. [24] reported association between the alpha-1 exponent and the sympathetic nervous system through direct measurements of sympathetic activity from the peroneus nerve. We did not confirm the relationship between the short-term alpha-1 exponent and the sympathetic activity, since it was not performed any procedure to isolate the sympathetic, it would surely strengthen the impact of our findings. However, we investigated the LF in normalized units, which is indicated to reflect action of the vagal and sympathetic activity on the heart, with a predominance of the sympathetic system [21]. We evaluated only female healthy volunteers. We should be careful when interpreting these data if applied in different population. Our study presents important findings to contribute to cardiovascular examinations in the clinical routine [27,28].

## Conclusion

The results indicate that the PCM induces an increase in the properties of short-term fractal correlations of heart rate dynamics accompanied with a decrease in the cardiac parasympathetic and global components of HRV analyzed through the time and frequency-domains of HRV. Moreover, fractal analysis of HRV was more sensitive to the classical spectral and time-domain analysis of HRV.

## Competing interests

The authors declare that they have no competing interest.

## Authors’ contributions

All authors participated in the design of the study and writing the manuscript as well as approving the final manuscript.
